# Paliperidone extended release for the treatment of pediatric and adolescent patients with Tourette's disorder

**DOI:** 10.1186/1744-859X-13-13

**Published:** 2014-05-02

**Authors:** Kazuhiko Yamamuro, Manabu Makinodan, Toyosaku Ota, Junzo Iida, Toshifumi Kishimoto

**Affiliations:** 1 Department of Psychiatry, Nara Medical University School of Medicine, 840 Shijo-cho, Kashihara, Nara 634-8522, Japan; 2 Faculty of Nursing, Nara Medical University School of Medicine, Kashihara 634-8522, Japan

**Keywords:** Tourette's disorder, Paliperidone extended release, Haloperidol, Tics

## Abstract

**Objective:**

A subgroup of patients with Tourette's disorder (TD) has symptoms refractory to haloperidol, a standard therapeutic drug for TD.

**Methods:**

We report on three cases of pediatric and adolescent patients who were treated with paliperidone extended release.

**Results:**

In two cases, TD symptoms were remarkably improved by switching from haloperidol to paliperidone extended release, and in another case, paliperidone extended release showed significant efficacy in treating TD symptoms as the first-line drug. In all cases, no significant adverse side effects were detected.

**Conclusion:**

Paliperidone extended release may be a strong candidate for the treatment of pediatric and adolescent patients with TD.

## Introduction

Tourette's disorder (TD) is a neurodevelopmental disorder commonly associated with the presence of multiple vocal and/or motor tics. The onset of TD occurs in childhood and the prevalence is higher in males than in females (4.3: 1) [1,2]. Of school-aged children, 6%–20% experience transient tics and 0.5%–1% suffer from chronic tics or TD [3]. TD usually has a familial component [2]. The majority of patients with TD also meet the criteria for one or more comorbid psychiatric disorders, including obsessive-compulsive disorder (OCD), attention-deficit/hyperactivity disorder (ADHD), mood disorder, and non-OCD anxiety disorder [4]. Since there are several biological hypotheses relating to TD that highlight dopaminergic function, typical antipsychotics such as haloperidol and pimozide have been prescribed to control tic symptoms [5]. More recently, clinical opportunities for prescribing atypical antipsychotics such as risperidone, quetiapine, aripiprazole, and olanzapine have increased owing to the enhanced efficacy and more tolerable side effect profiles relative to classical antipsychotics [1,6-8]. Considering all antipsychotics, risperidone is commonly recommended by experts [9,10].

This report focuses on the utilization of the atypical antipsychotic, paliperidone extended release (ER), which chemically is a major active metabolite of risperidone (9-hydroxyrisperidone), in the treatment of child and adolescent patients with TD since our survey of the literature has failed to identify any current reports related to its use in this setting. The three cases showed reductions in TD symptom severity over a relatively short time period and an improvement in the Yale Global Tic Severity Scale (YGTSS) [11], a clinician-rated, semistructured interview useful for determining both the effects of treatment as well as providing an assessment of tic severity (0–50 scale range, impairment score not included). Motor and phonic tics were rated separately according to number, frequency, intensity, complexity, and interference.

According to the World Medical Association Declaration of Helsinki, a statement of ethical principles for medical research in human patients, we provided patients and patients’ parents with thorough monitoring information and any serious adverse events.

## Case presentation

### Case A

Patient A is a 10-year-old boy who developed facial motor tics such as blinking and grimacing at 8 years of age. He also began to express involuntary utterances consisting of the vowel sound ‘a’ and excessive shoulder shrugging when he was 9 years old. At the age of 10, the abnormal ‘a’ vocalization was replaced with very loud snoring. Subsequently, he began to display involuntary coprolalia using words such as ‘kill’ and ‘die.’ Treatment with haloperidol (1.5 mg/day) failed to improve the tic symptoms while being followed at a local clinic. Since the tics had a significant negative impact on his activities of daily living and social relationships, he was referred to our hospital when he was 10 years old. TD was diagnosed in accordance with DSM-IV-TR and his YGTSS was 27. He did not have ADHD, OCD, or other behavioral or psychiatric disorders. He was prescribed with 3 mg of paliperidone ER to improve tic symptoms and scheduled for weekly visits to our hospital to monitor side effects. One week after initiation of paliperidone ER, the occurrence of coprolalia had dramatically improved, although he still displayed motor and vocal tics (YGTSS 20), which were gradually improved with few side effects. Within 2 months, his vocal tic symptoms had nearly disappeared, but slight shoulder shrugging remained (YGTSS 7).

### Case B

Patient B is an 11-year-old boy who developed motor tics such as blinking and coughing at 9 years old. He began to express an involuntary vocal utterance as a very loud ‘a’ sound at the age of 11. Since both the frequency and severity of these motor and vocal tics worsened, he was referred to our hospital. His was diagnosed with TD in accordance with DSM-IV-TR and his YGTSS was 21. He did not have ADHD, OCD, or other behavioral or psychiatric disorders. First, he was prescribed with 3 mg/day of paliperidone ER to improve the tic symptoms. While the volume of his vocal tics became lower after three weeks of paliperidone ER treatment, his overall tic symptoms were not improved (YGTSS 17). After having been on 3 mg/day of paliperidone ER for 6 weeks, his dosage was increased to 6 mg/day. Two weeks after initiating the 6 mg/day paliperidone ER treatment, both the motor and vocal tic symptoms had substantially improved with few side effects (YGTSS 7).

### Case C

Patient C is 13-year-old boy who developed motor tics such as violent neck shaking, repeated jumping, and sniffing as well as vocal tics beginning at 8 years of age. He visited our hospital when he was 11 years old and was diagnosed with TS in accordance with DSM-IV-TR and his YGTSS was 18. While there was a family history of TD, neither ADHD, OCD, nor other behavioral or psychiatric disorders were identified. He had been treated with haloperidol (1.5 mg/day) for approximately 2 years and showed only mild improvement while the presence of drowsiness prevented further dosage titration. He was prescribed with 3 mg/day of paliperidone ER and showed no improvement in his tic symptoms over the course of 3 weeks following initiation of therapy (YGTSS 16). Subsequently, the dosage of paliperidone ER was increased to 6 mg/day. After 5 weeks of higher dosage of paliperidone ER, he did experience mild drowsiness but significant improvement of the tic symptoms was observed (YGTSS 6). Due to the presence of the mild drowsiness, the dosage of paliperidone ER was then reduced to 3 mg/day and, despite this reduction, his tic symptoms did not recur over the following 4 months (YGTSS 7).

## Discussion

To the best of our knowledge, only a single case study has reported beneficial effects of paliperidone ER for the treatment of an adult patient who was diagnosed with TD and comorbid schizophrenia [12]. The cases presented here are the first reports showing the therapeutic effects of paliperidone ER in treating child and adolescent patients with TD.

While the exact pathobiology of TD remains unknown, several hypotheses have been proposed. Dysfunction in the cortico-striatal-thalamo-cortical (CSTC) circuits has been suggested as a potential cause of TD [13]. Previous studies using single-photon emission computed tomography (SPECT) have suggested higher levels of dopamine transporter binding in the caudate and putamen nuclei in TD patients as compared to healthy controls [14]. Furthermore, greater putamen dopamine release was observed in TD in comparison to healthy controls using positron emission tomography (PET) [15]. These findings are supportive of abnormalities in dopaminergic function as potential participants in the pathophysiology of TD. Therefore, typical or atypical antipsychotics, which generally block dopaminergic signaling, have been prescribed to control its tic symptoms. Of all antipsychotics, currently risperidone is recommended by the experts to treat tic symptoms [9,10]. Risperidone has potent dopamine-2 (D_2_) and 5-hydroxytryptamine (5-HT2A) receptor blocking properties, and it has been proven to be as effective for the treatment of TD as haloperidol and relatively safer compared to haloperidol in terms of side effects (e.g., high frequency of extrapyramidal symptoms by haloperidol) [16,17]. While the major anti-tic efficacy of risperidone is probably due to the blocking of dopaminergic neurotransmission, the serotonergic action might give additional effects by indirectly attenuating mesolimbic and/or mesocortical dopaminergic pathways [18]. Therefore, risperidone could be a better pharmacotherapeutic agent for the treatment of TD than other typical antipsychotics such as haloperidol. However, resperidone also has its own problematic side effects including oversedation and weight gain, and here we would like to suggest the possibility that paliperidone ER, the extended-release form of the major metabolite of risperidone, could be used to avoid these adverse events especially since blood concentration of paliperidone ER is relatively stable [19] leading to less daytime somnolence [20] and it can produce fewer extrapyramidal symptoms as compared to risperidone [21]. As daytime somnolence and extrapyramidal symptoms substantially disturb children's life; study, exercise, friendship, and so on, paliperidone ER may serve as an advantageous pharmacotherapy for the treatment of child and adolescent TD.

## Conclusion

These cases suggest that paliperidone ER might serve as an efficacious therapy in child and adolescent patients with TD and present few side effects. Further information and details provided by studies using larger sample sizes are needed to validate the apparent efficacy, safety, and tolerability of paliperidone ER in the treatment of child and adolescent patients with TD.

## Consent

Written informed consent was obtained from the patient's parents for the publication of this report and any accompanying images.

## Abbreviations

ADHD: attention-deficit/hyperactivity disorder; OCD: obsessive-compulsive disorder; TD: Tourette's disorder; YGTSS: Yale Global Tic Severity Scale.

## Competing interests

The authors declare that they have no competing interests.

## Authors' contributions

KY was involved in the collection of the data and wrote the first draft of the manuscript. MM, TO, JI and TK supervised the entire project and was critically involved in the design, and contributed to the editing of the final manuscript. All authors have read and approved the final manuscript.
